# Mixed lipopeptide-based mucosal vaccine elicits a long-term bone marrow memory response that is potentially cross-reactive against a broad-spectrum of coronaviruses in mice

**DOI:** 10.3389/fimmu.2025.1619882

**Published:** 2025-07-28

**Authors:** Raj S. Patel, Babita Agrawal

**Affiliations:** Department of Surgery, Faculty of Medicine and Dentistry, College of Health Sciences, University Alberta, Edmonton, AB, Canada

**Keywords:** SARS-CoV-2, lipopeptide-based vaccine, mucosal immunity, pan-coronavirus, broad-spectrum, immunological memory

## Abstract

**Introduction:**

SARS-CoV-2 is continuing to prevail as an endemic virus, and therefore, we need a next-generation vaccine that prevents SARS-CoV-2 infections, broadly protects against multiple CoVs, and induces long-term local and systemic immunity. To address that need, we have designed a mixed lipopeptide-based pan-coronavirus (LP_Mix_) vaccine based on T and B cell epitopes derived from highly conserved and functional regions of the SARS-CoV-2 spike (S), nucleocapsid (N), and membrane (M) proteins.

**Methods:**

Male C57BL/6 mice (n=5 per group) were immunized intranasally twice, 14 days apart, with the LP_Mix_ vaccine candidates, which consisted of seven lipopeptides (LP1–LP7), with or without HKCC (heat-killed *Caulobacter crescentus*), a novel mucosal adjuvant. At 2.5 weeks, 2 months, and 7 months post-immunization, lung, spleen, bone marrow, and bronchoalveolar lavage (BAL) samples were collected for immunological analyses. Additionally, blood samples were collected monthly to monitor antibody titers.

**Results:**

We demonstrate that intranasal immunizations of mice with LP_Mix_ induced a long-lasting systemic IgM/IgG, and mucosal IgA response against a broad-spectrum of CoVs, showing clinically significant levels of neutralizing antibody titers. Splenocytes and bone marrow cells, derived from LP_Mix_ immunized mice, demonstrated a robust proliferation response against vaccine antigens (P_1-7_), which were maintained up to 2 months and 7 months, after LP_Mix_ immunizations, respectively. Moreover, antigen-specific B cells and memory CD4^+^/CD8^+^ T cells were long-lived and maintained up to 7 months after LP_Mix_ immunizations, in the lungs, spleen and bone marrow. The addition of HKCC (heat-killed *Caulobacter crescentus*), a novel mucosal adjuvant, promoted the longevity of memory CD4^+^/CD8^+^ T cell and B cell responses.

**Discussion:**

Overall, our study demonstrates that a mucosal lipopeptide-based vaccine targeting conserved SARS-CoV-2 epitopes elicits durable, long-lasting immune responses against a broad range of coronaviruses.

## Introduction

Severe acute respiratory syndrome coronavirus 2 (SARS−CoV−2), the causative agent of coronavirus disease 2019 (COVID-19), has caused more than 775 million infections and over 7 million deaths worldwide (1). Although the COVID-19 pandemic is no longer considered a public health emergency, the SARS-CoV-2 virus persists as an endemic respiratory virus with the potential to cause future pandemics (2). The development of an effective, long-lasting, and preventive vaccine for SARS-CoV-2 has been a challenge as immunity acquired from natural infections and/or vaccines is incomplete and transient. During the COVID-19 pandemic, the 1^st^ generation vaccines were highly efficacious (exceeding 94%), protecting against hospitalizations, severe disease outcomes, and deaths (3). However, these Spike (S)-based vaccines induced short-lived, variant-specific immunity that lacked protection at the lung mucosal barrier and do not prevent SARS-CoV-2 infections. Furthermore, the current approach of periodically updating vaccine constructs with the S-protein of the latest circulating SARS-CoV-2 variant has created a cycle of administering repeated booster shots. With the current vaccination program against SARS-CoV-2, issues related to vaccine hesitancy, antigenic sin, back-boosted immunity, immune tolerance, and inflammation have necessitated the need for a next-generation COVID-19 vaccine that enhances protection against a broad-spectrum of CoVs, prevents SARS-CoV-2 infections, and induces durable, long-term protective immunity (4–7).

Many successful vaccines have been developed against respiratory viruses, including smallpox, varicella zoster virus (VZV), measles, mumps, and rubella, that induce life-long, preventive immunity which leads to their elimination or eradication (2). Although these are all live attenuated viral vaccines, learning the principles and correlates of protective immunity from these successful vaccines can help develop the next-generation vaccine for COVID-19. These vaccines are able to stimulate multiple immune compartments (including the local mucosal tissue, systemic blood and spleen), and various innate and adaptive immune cell types (2). Many human and experimental animal models have shown the presence of memory T cells, antibody-secreting B cells, and IgA in the mucosal-associated lymphoid tissue (MALT) that help in controlling respiratory infections (8–10). Similarly, high levels of circulating IgM/IgG in the blood, neutralizing antibody (nAb) titers, and the induction of clonally-expanded B and T lymphocytes are indicators of a well-established adaptive immune response (2). Generally, vaccines with longer half-lives allow for better exposure, cross-talk, and coordination between immune compartments including the upper and lower respiratory tracts, and the systemic immune system (11). Moreover, they enhance antigen sensing, presentation, and activation of downstream effector functions (11). Finally, vaccines that successfully control respiratory viral infections elicited life-long protective immunity (2). Notably, lipopeptide-based vaccines have inherent characteristics that facilitate the induction of a comprehensive adaptive response, including their ability to self-assemble into nano-/micro-particles, passively crossing cellular and mucosal membranes, efficiently delivering vaccine antigens to MALTs, enhance antigen presentation on major histocompatibility complexes (MHC), activate antigen-presenting cells (APCs), and induce local and systemic epitope-specific immune responses (12–14). In addition, lipopeptide constructs are stable with fewer storage restrictions, are cost-effective and are easily scalable, making this type of vaccine more accessible around the world (3, 12–14). Interestingly, in humans and several experimental animal models, lipopeptide-based vaccines have induced long-term immune responses against multiple pathogens, including influenza, *Plasmodium falciparum*, HBV, and HIV (15–19). With regards to SARS-CoV-2, the SpFN + ALFQ and MCMV-based wild-type spike-based vaccine induced long-lasting protective immunity (20–22). Understanding why certain vaccines induce life-long immunity whereas others do not, remains an unresolved area of investigation. Many clinical studies have shown that immune responses generated against highly conserved SARS-CoV-2 N-/M-proteins are long-lasting, broaden the coverage of variants and lead to protection against severe disease outcomes (23–26). Therefore, it is important to incorporate N-/M-proteins in the next-generation vaccine design. Furthermore, the bone marrow has been shown as a compartment for maintaining long-lasting, functional memory B and T lymphocytes (27). Specialized survival niches, established by resident stromal cells in the bone marrow, facilitate the longevity and self-renewal of memory immune cells for a lifetime (28–32). Perhaps, the ability of a vaccine to induce long-lasting immunity may be best evaluated by investigating long-term vaccine-induced responses in bone marrow.

We have identified seven highly conserved, immunodominant epitopes (P_1-7_) from the SARS-CoV-2 S-, N-, and M-proteins, based on their promiscuous binding of MHC class I/II molecules, CD4^+^/CD8^+^ T cell and B cell epitope predictions, antigenic propensity, surface exposure, and conservativity scoring (24, 25, 33–43). All seven epitopes were incorporated into a synthetic lipopeptide vaccine construct, which consists of a peptide conjugated with a palmitoyl moiety at the carboxyl terminus and the seven conjugates combined to formulate our lipopeptide-based vaccine (43). Furthermore, the LP_Mix_ vaccine with or without a mucosal adjuvant HKCC (heat-killed *Caulobacter crescent*), induced potent immune responses in mice, and was efficacious in protecting against lung pathology in hamsters infected with SARS-CoV-2 Omicron (BA.5) (44).

In this study, we sought to investigate whether our LP_Mix_ vaccine formulations induce long-term immunity against a broad-spectrum of coronavirus spike proteins and the SARS-CoV-2 N- and M-proteins, in various compartments of the immune system including the lungs, blood and spleen, and bone marrow. Our studies demonstrated that two intranasal immunizations with LP_Mix_ elicited a systemic IgM/IgG and mucosal IgA response against S-proteins of seven SARS-CoV-2 VOCs, SARS-CoV-1 (Tor2), MERS-CoV, and two HCoVs, and the SARS-CoV-2 N-/M-proteins, for up to 7 months. In addition, measurable and clinically-relevant nAb titers were induced and maintained for up to 2 months. Moreover, there was a long-lasting presence of antigen-specific B cells, and memory CD4^+^/CD8^+^ T cells in the bone marrow of mice, supporting the establishment of sustained memory responses upon LP_Mix_ immunizations. Incorporation of HKCC adjuvant to LP_Mix_ vaccine bolstered the mucosal IgA, and nAb responses, and longevity of these responses. The results reported here could pave the way to investigating mucosal pan-coronavirus vaccines inducing protracted immunity.

## Results

### Intranasal immunizations with LP_Mix_ induced systemic IgM/IgG and mucosal IgA responses against multiple SARS-CoV-2 VOCs that were maintained up to 7 months

**Figure 1:**
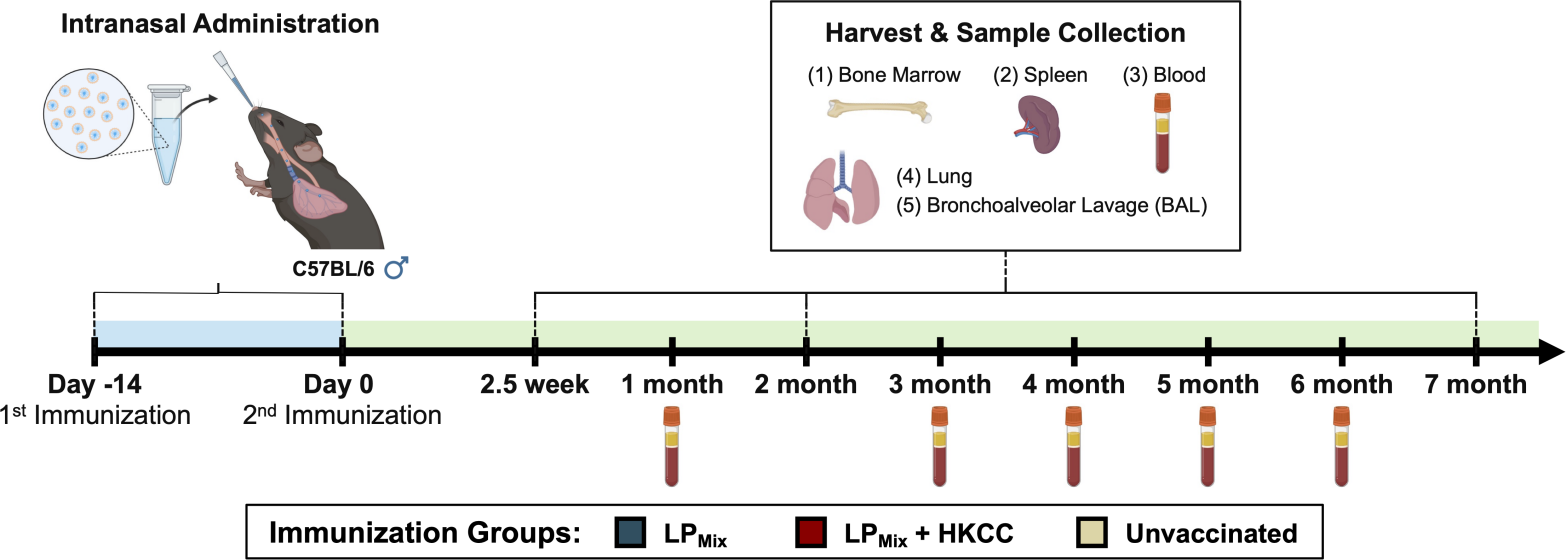
Vaccine regimen and sample collection timeline for long-term immunological analysis. Male C57BL/6 mice (n=5 per immunization group) were immunized (D-14, D0) intranasally with a lipopeptide mix (LP_Mix_) vaccine formulation, consisting of seven lipopeptides (LP_1_- LP_7_), each administered at 10 µg/mouse, in the presence and absence of HKCC adjuvant. At 2.5 weeks, 2 months, and 7 months, bone marrow, spleen, lung, and BAL samples were collected for immunological analysis. Additionally, blood samples were collected every month.

The design, characterization, and immunogenicity of the lipopeptide mix (LP_Mix_) vaccine construct with the HKCC mucosal adjuvant has been previously described (44). Male C57BL/6 mice (n=5/immunization group) were intranasally immunized (D-14, D0) with an LP_Mix_ vaccine formulation, and various immunological studies were carried out from 2.5 weeks to 7 months after the 2^nd^ immunization (**Figure 1**).

**Figure 2:**
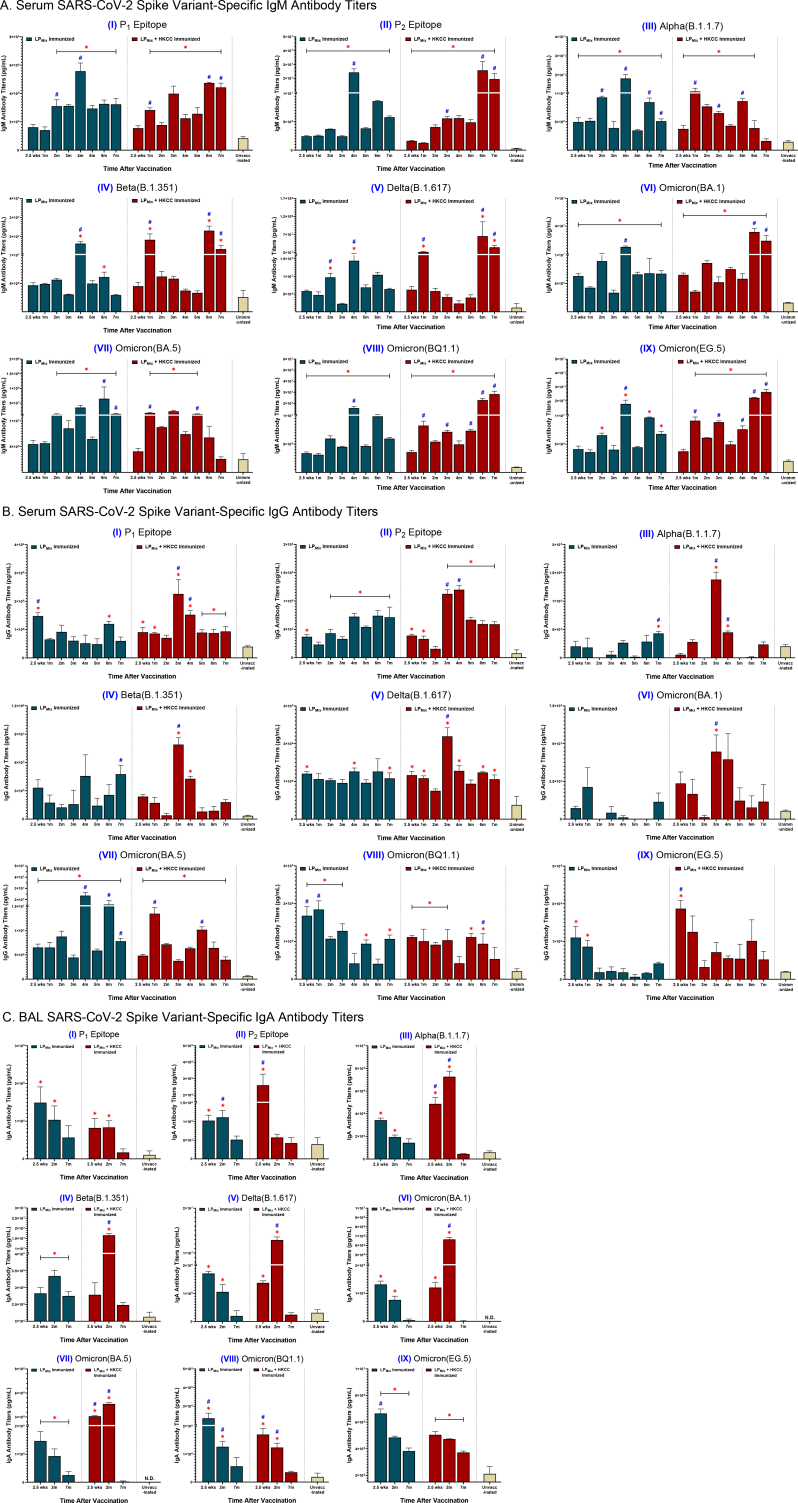
LP_Mix_ immunizations induce long-lasting SARS-CoV-2 spike variant-specific antibody titers. Male C57BL/6 mice (n=5 per immunization group) were immunized (D-14, D0) intranasally with LP_Mix_ groups as described in **Figure 1**. After the 2^nd^ immunization, serum samples were collected on a monthly basis, up to 7 months. BAL samples were collected at 2.5 weeks, 2 months, and 7 months, after the 2^nd^ immunization. **(A)** Serum IgM, **(B)** serum IgG, and **(C)** BAL IgA antibody titers were measured against the SARS-CoV-2 S-epitopes, (I) P1 and (II) P2, and S-proteins of the (III) Alpha (B.1.1.7), (IV) Beta (B.1.351), (V) Delta (B.1.617), (VI) Omicron (BA.1), (VII) Omicron (BA.5), (VIII) Omicron (BQ1.1), and (IX) Omicron (EG.5) variants. Bars represent mean [Ig] ± SEM of triplicate wells, from two independent repeat experiments. Statistical significance was determined using a two-way ANOVA, followed by Tukey’s *post-hoc* test. * and # indicated significant differences (P ≤ 0.05) between the LP_Mix_-immunized and unvaccinated groups, and the respective adjuvant and non-adjuvant groups, respectively.

Following LP_Mix_ immunizations, serum IgM/IgG and BAL IgA titers were evaluated against the SARS-CoV-2 S-epitopes, P_1_ and P_2_, and S-proteins of the Alpha (B.1.1.7), Beta (B.1.351), Delta (B.1.617), and Omicron (BA.1, BA.5, BQ1.1, EG.5) variants, for up to 7 months (**Figure 2**). Tracking serum antibody titers over this period we observed that the IgM and IgG titers fluctuated in a wave-like pattern, with antibody titers peaking at different timepoints and the magnitude of those peaks varying over time, depending on the LP_Mix_ immunization group and the SARS-CoV-2 variant tested (**Figures 2A, B**).

Immunizations with LP_Mix_ showed serum IgM titers peaked at 2, 4, and 6 months against the P_2_ epitope, and the SARS-CoV-2 Alpha, Beta, Delta, and Omicron (BA.5, BQ1.1, EG.5) variants (**Figures 2A**, II-V, VII-IX). Furthermore, LP_Mix_ immunizations demonstrated serum IgM titers peaked at 2 and 4 months for the P_1_ epitope, and at 2.5 weeks, 2 and 4 months for the Omicron (BA.1) variant (**Figures 2A**, I, VI). Immunizations with LP_Mix_ and HKCC adjuvant induced increasing serum IgM titers against the P_1_ and P_2_ epitopes, and the SARS-CoV-2 Beta, Delta, and Omicron (BA.1, BQ1.1, EG.5) variants fluctuated in a wave pattern over time (**Figures 2A**, I, II, IV-VI, VIII, IX). In contrast, serum IgM titers against the Alpha and Omicron (BA.5) variants showed a declining trend after peaking at 5 months (**Figures 2A**, I, V). All in all, LP_Mix_ immunizations induced multiple waves of serum IgM antibodies, and the addition of HKCC adjuvant led to increasing antibody-peaks over time with each successive wave.

LP_Mix_ immunizations induced serum IgG titers that were initially high, followed by a decline over the 7 months against the SARS-CoV-2 Omicron (BQ1.1, EG.5) variants (**Figures 2B**, VIII, IX). In contrast, with the exception of IgG titers in the 3^rd^ and 4^th^ month, LP_Mix_-induced serum IgG titers against the SARS-CoV-2 Alpha, Beta, and Omicron (BA.1) variants were comparable to the unvaccinated groups (**Figures 2B**, III, IV, VI). Interestingly, LP_Mix_-induced serum IgG recognized the Delta variant, and the Ig levels were maintained over the 7 months (**Figures 2B**, V). The serum IgG titers against the Omicron (BA.5) variant fluctuated over the 7 months, with peaks at 2, 4, and 6 months (**Figures 2B**, VII). Next, LP_Mix_ + HKCC induced serum IgG titers that declined from 2.5 weeks to 2 months, followed by a spike at 3 months, and declined thereafter, against the P_1_ epitope, and the SARS-CoV-2 Alpha, Beta, Delta, and Omicron (BA.1) variants (**Figures 2B**, I-VI). Similarly, serum IgG titers against the P_2_ epitope showed an additional spike in the 4^th^ month before titers started to decline (**Figures 2B**, II). Moreover, IgG titers against the Omicron (BQ1.1) variant were maintained up to 5^th^ month before declining (**Figures 2B**, VIII). Likewise, serum IgG titers against the Omicron (EG.5) variant were initially high, followed by a decline over the 7 months (**Figures 2B**, IX). Lastly, against the Omicron (BA.5) variant, LP_Mix_ + HKCC immunization induced serum IgG titers that peaked at the 1 and 5 month timepoints, showing well-defined, wave-like characteristics (**Figures 2B**, VII). Altogether, serum IgG titers induced upon LP_Mix_ immunizations reacted against all SARS-CoV-2 variants, but they were more prominent and persistent against the Delta (B.1.617), and Omicron (BA.5, BQ1.1) variants, compared to the unvaccinated groups.

BAL IgA titers were measured to investigate the induction of mucosal immunity. LP_Mix_ immunizations induced IgA titers against the P_1_ epitope, and the SARS-CoV-2 Alpha (B.1.1.7), Delta (B.1.617), and Omicron (BA.1, BA.5, BQ1.1, EG.5) variants that peaked at 2.5 weeks and declined thereafter (**Figures 2C**, I, III, V-IX). In contrast, LP_Mix_-induced BAL IgA titers against the P_2_ epitope and Beta variant that peaked at 2 months, followed by a decline at 7 months (**Figures 2C**, II, IV). Likewise, LP_Mix_ + HKCC immunizations induced IgA titers that peaked at 2 months, followed by a decline at 7 months, against the P_1_ epitope, and multiple SARS-CoV-2 variants including the Alpha, Beta, Delta, and Omicron (BA.1, BA.5) variants (**Figures 2C**, I, III-VII). Whereas, LP_Mix_ + HKCC-induced BAL IgA titers against the P_2_ epitope and the Omicron (BQ1.1, EG.5) variants demonstrated a declining trend over time (**Figures 2C**, II, VIII, IX). Considering cross-reactive IgA responses against all SARS-CoV-2 variants except for Omicron (BQ1.1) and Omicron (EG.5), IgA responses induced upon LP_Mix_ immunizations declined over time, whereas the addition of HKCC adjuvant led to IgA responses that peaked at 2 months, followed by a decline at 7 months (**Figure 2C**).

### LP_Mix_ immunizations induced Omicron(BA.1)-specific IgM^+^ B cells in the lungs, spleen, and bone marrow at 2.5 weeks, 2 months, and 7 months

The recruitment, activation, and maturation of antigen-specific B cells is essential for an effective humoral response (45). Here, we assessed the cell counts and MFIs (mean fluorescent intensities) of Omicron(BA.1)-specific IgM^+^ B cells in response to LP_Mix_ immunizations.

**Figure 3:**
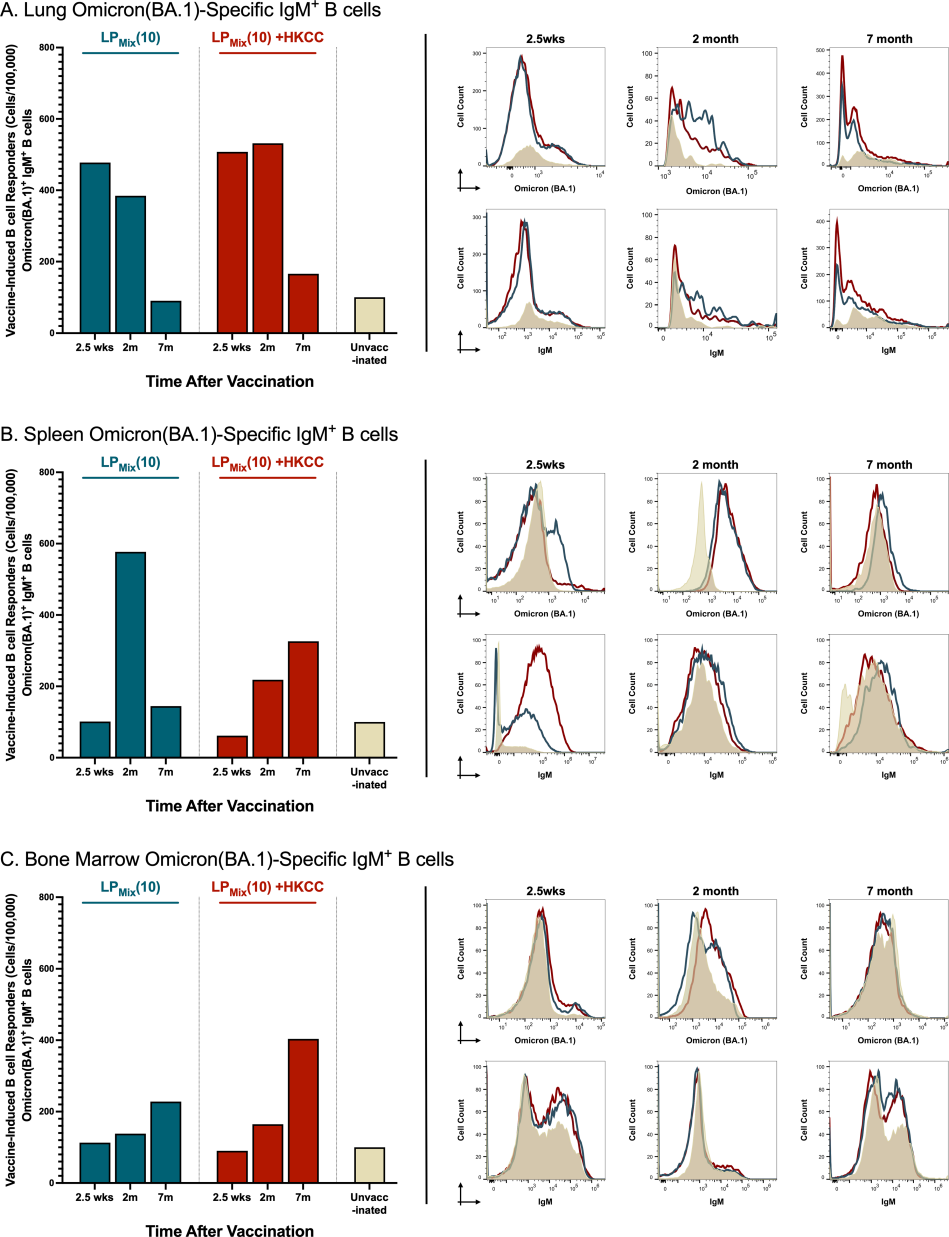
LP_Mix_ immunizations induce long-lasting Omicron(BA.1)-specific IgM^+^ B cells in the lungs, spleen, and bone marrow. Male C57BL/6 mice (n=5 per immunization group) were immunized (D-14, D0) intranasally with LP_Mix_ groups as described in **Figure 1**. At the 2.5 week, 2 month, and 7 month timepoint, the **(A)** lungs, **(B)** spleen, and **(C)** bone marrow were harvested. Using flow cytometry, the cell counts of Omicron(BA.1)-specific IgM^+^ B cells were determined. Bar graphs summarized the mean number of Omicron (BA.1)^+^ IgM^+^ B cells, among LP_Mix_ immunization groups. Data were represented as the percent of unvaccinated control, for each LP_Mix_ immunization group, for their respective timepoints. Histograms showed the B cell surface immunoglobulin (Ig) binding to the Omicron (BA.1) S-protein, and the expression of surface IgM on Omicron(BA.1)-specific IgM^+^ B cells. Data shown is reflective of two independent, repeat flow cytometry experiments.

Consistent with the BAL IgA responses, LP_Mix_ immunizations induced lung Omicron(BA.1)-specific IgM^+^ B cell counts that decreased over the period of 2.5 weeks to 7 months (**Figure 3A**). However, in the spleen, Omicron(BA.1)-specific IgM^+^ B cell counts expanded from 2.5 weeks to 2 months, followed by a contraction of these B cell numbers at 7 months (**Figure 3B**). Interestingly, in contrast to mucosal and systemic antigen-specific B cells, Omicron(BA.1)-specific IgM^+^ B cell counts steadily increased over the 7 months in the bone marrow (**Figure 3C**).

Furthermore, LP_Mix_ + HKCC immunizations induced lung Omicron(BA.1)-specific IgM^+^ B cells that were maintained up to 2 months, followed by a decline at 7 months (**Figure 3A**). Moreover, in the spleen and bone marrow, Omicron(BA.1)-specific IgM^+^ B cell counts increased over time (**Figures 3B, C**).

### LP_Mix_ immunizations induced cross-reactive IgM, IgG and IgA responses against heterologous CoVs

To determine the potential of LP_Mix_ as a pan-coronavirus vaccine, serum and BAL samples collected at various timepoints (described in **Figure 1**) were used to measure antibody binding to Spike proteins from heterologous CoVs, including HCoV-OC48, HCoV-299E, MERS-CoV, and SARS-CoV-1 (Tor2).

**Figure 4:**
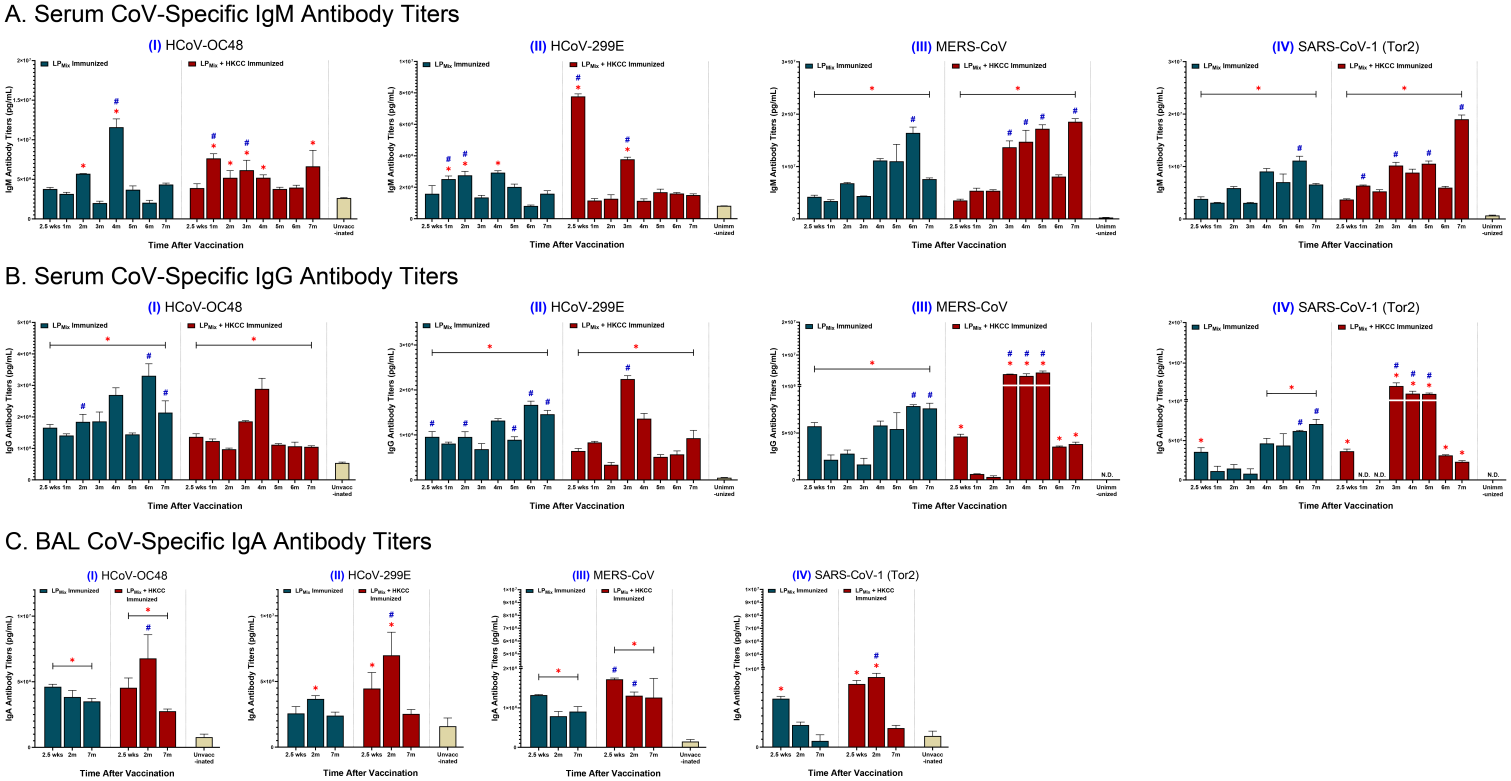
Cross-reactive antibody titers induced against heterologous CoVs by intranasal immunizations with LP_Mix_ groups. Male C57BL/6 mice (n=5 per immunization group) were immunized (D-14, D0) intranasally with LP_Mix_ groups as described in **Figure 1**. After the 2^nd^ immunization, serum samples were collected on a monthly basis, up to 7 months. BAL samples were collected at 2.5 weeks, 2 months, and 7 months, after the 2^nd^ immunization. **(A)** Serum IgM, **(B)** serum IgG, and **(C)** BAL IgA antibody titers were measured against the S-proteins of heterologous CoVs, including (I) HCoV-OC48, (II) HCoV-299E, (III) MERS-CoV, and (IV) SARS-CoV-1 (Tor2). Bars represent mean [Ig] ± SEM of triplicate wells, from two independent repeat experiments. Statistical significance was determined using a two-way ANOVA, followed by Tukey’s *post-hoc* test. * and # indicated significant differences (P ≤ 0.05) between the LP_Mix_-immunized and unvaccinated groups, and the respective adjuvant and non-adjuvant groups, respectively.

IgM titers against HCoV-OC48 showed multiple waves of antibodies, which peaked at the 2, 4, and 7 month timepoints (**Figures 4A**, I). Next, over the 7 months, IgM titers against HCoV-299E were similar to the unvaccinated group, except at the 1, 2, and 4 month timepoints (**Figures 4A**, II). Lastly, IgM titers against MERS-CoV and SARS-CoV-1 (Tor2) increased over time, with peaks at 2, 4, and 6 months (**Figures 4**III, IV). Moreover, for the LP_Mix_ + HKCC immunization group, IgM titers against HCoV-OC48 were significantly higher compared to the unvaccinated group, from the 1 month to the 4 month timepoints, and at 7 months (**Figures 4A**, I). Also, IgM titers against HCoV-299E declined over time, with significantly higher antibody peaks at 2.5 weeks, and 3 months (**Figures 4A**, II). With regards to MERS-CoV and SARS-CoV-1(Tor2), LP_Mix_ + HKCC immunization group showed increased IgM titers over time, with peaks at 3, 4, 5, and 7 months, and at 1, 3, 5, and 7 months, respectively (**Figures 4**III, IV). Overall, LP_Mix_ immunizations induced serum IgM titers that fluctuated over time, with antibody peaks gradually increasing over time against MERS-CoV and SARS-CoV-1 (Tor2), and decreasing against HCoV-299E.

Next, LP_Mix_ elicited IgG titers against HCoV-OC48 and -299E that increased over time, with peaks in the 4^th^ and 6^th^ month (**Figures 4B**, I, II). Also, LP_Mix_-induced IgG titers against MERS-CoV and SARS-CoV-1(Tor2) declined from 2.5 weeks to 3 months, followed by a gradual increase in titers up to 7 months (**Figures 4B**, III, IV). The LP_Mix_ + HKCC immunizations demonstrated a similar pattern of serum IgG titers against most heterologous CoVs—characterized by a decline in titers from 2.5 weeks to 2 months, followed by an increase in titers starting from the 3^rd^ month, and a decline in the 6^th^ and/or 7^th^ months (**Figures 4B**, I-IV). Altogether, LP_Mix_ + HKCC induced serum IgG titers against heterologous CoVs that peaked between 3–5 months after the 2^nd^ immunization.

Looking at mucosal IgA responses, LP_Mix_ immunizations induced IgA titers against HCoV-OC48, HCoV-299E, MERS-CoV, and SARS-CoV-1 (Tor2) that declined over time (**Figures 4C**, I-IV). The LP_Mix_ + HKCC group induced IgA titers peaked at 2 months, followed by a decline at 7 months, against HCoV-OC48, HCoV-299E, and SARS-CoV-1 (Tor2), whereas, IgA titers against MERS-CoV were maintained up to 7 months (**Figures 4C**, I-IV).

### LP_Mix_ immunizations induced systemic and mucosal NAb titers that were sustained up to 7 months

NAb titers strongly correlate with protection against SARS-CoV-2 infections and disease outcomes (46). Here, we tested serum and BAL samples collected from LP_Mix_ immunization groups for their ability to block spike-ACE2 interactions over a period of 7 months. Consistent with the antibody binding to various spike antigens (**Figures 2**, **3**), nAb titers also demonstrated a wave like pattern over the period of 7 months.

**Figure 5:**
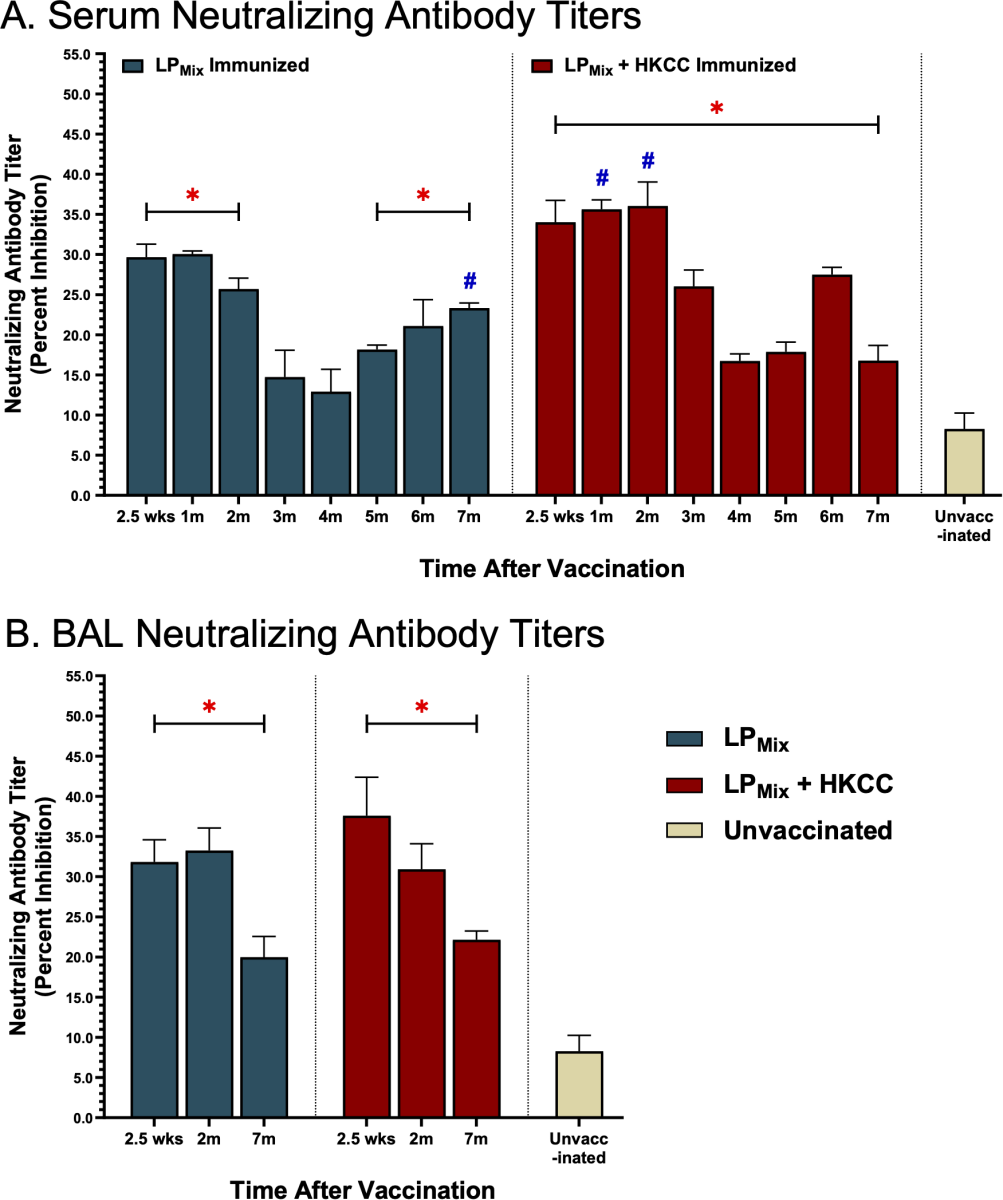
Intranasal immunizations with LP_Mix_ groups induce sustained neutralizing antibody titers in the serum and lung mucosa. Male C57BL/6 mice (n=5 per immunization group) were immunized with LP_Mix_ groups, twice, 14 days apart. Serum and BAL samples were collected at various timepoints, as described in **Figure 1**, to determine neutralizing antibody titers. **(A)** Serum and **(B)** BAL neutralizing antibody titers were expressed as percent inhibition. Data was shown as mean ± SEM of triplicate wells, from two independent repeat experiments. Two-way ANOVA, followed by Šidák *post-hoc* test was used to determine significance. * and # indicated significant differences (P ≤ 0.05) between the LP_Mix_-immunized and unvaccinated groups, and the respective adjuvant and non-adjuvant groups, respectively.

From 2.5 weeks to 2 months, all LP_Mix_ immunizations induced serum NAb titers that were well-above the clinically significant threshold of 15% (**Figure 5A**) (47). However, by the 3^rd^ and 4^th^ month, serum NAb titers declined by approximately 10-20%. Interestingly, LP_Mix_ immunizations showed a gradual increase in NAb titers, starting from the 5^th^ month onwards (**Figure 5A**). In contrast, the addition of the HKCC adjuvant to LP_Mix_, led to overall increase in NAb titers that were sustainable for up to 7 months, with NAb titers peaking at 2 months, and fluctuating between the 4–6 months (**Figure 5A**). Overall, LP_Mix_ immunizations induced NAb titers that were clinically significant, and significant compared to the unvaccinated group (**Figure 5A**).

Next, looking at mucosal NAb titers, LP_Mix_ immunization with or without adjuvant HKCC, showed clinically significant BAL NAb titers, at all timepoints (2.5 weeks, 2 months, and 7 months) but the titer was reduced by 7 months (**Figure 5B**). Furthermore, all BAL NAb titers were significant compared to the unvaccinated group (**Figure 5B**).

### Intranasal immunizations with LP_Mix_ groups induced systemic and mucosal N-/M-specific antibody titers

**Figure 6:**
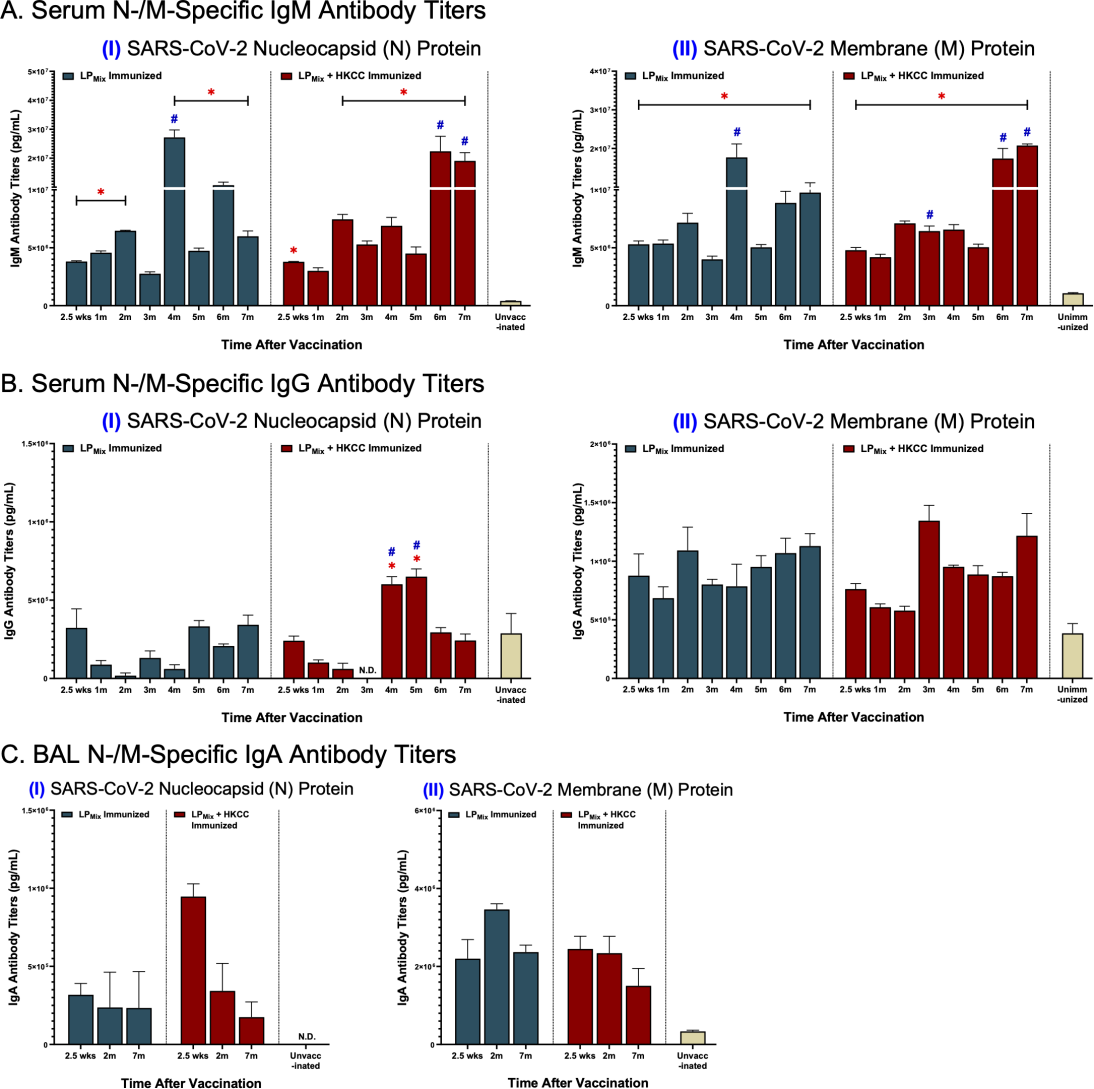
LP_Mix_ immunizations induce long-lasting systemic IgM/IgG and mucosal IgA antibody titers against the SARS-CoV-2 N-/M-proteins. Male C57BL/6 mice (n=5 per immunization group) were immunized with LP_Mix_ groups, twice, 14 days apart. Serum and BAL samples were collected at various timepoints, as described in **Figure 1**. **(A)** Serum IgM, **(B)** serum IgG, and **(C)** BAL IgA antibody titers were tested against the SARS-CoV-2 (I) N- and (II) M-proteins. Bars represent mean [Ig] ± SEM of triplicate wells, from two independent repeat experiments. Two-way ANOVA, followed by Tukey’s *post-hoc* test was used to determine significance. * and # indicated significant differences (P ≤ 0.05) between the LP_Mix_-immunized and unvaccinated groups, and the respective adjuvant and non-adjuvant groups, respectively.

Over a 7 month period, we examined antibody titers against the SARS-CoV-2 N-/M-proteins, from serum and BAL samples collected from LP_Mix_ and LP_Mix_ + HKCC immunized groups. IgM titers demonstrated a similar wave-like pattern as previously described. N-specific IgM titers peaked at 2, 4, and 6 months for both immunization groups (**Figures 6A**, I). Furthermore, IgM titers against the M-protein showed peak titers at 2, 4, and 7 months for the LP_Mix_ immunization group, whereas, they increased over time for the LP_Mix_ + HKCC immunization group (**Figures 6A**, II). Next, there were low levels of serum IgG titers induced by LP_Mix_ immunizations against the N-protein (**Figures 6B**, I). Whereas, LP_Mix_ immunizations induced M-specific IgG titers that increased over time (**Figures 6B**, II). Lastly, BAL IgA titers against the N-protein were detectable but remained low (**Figures 6C**, I). Only the LP_Mix_ + HKCC immunizations induced significantly elevated BAL IgA titers at 2.5 weeks, compared to the unimmunized group (**Figures 6C**, I). Next, LP_Mix_ immunizations induced M-specific IgA titers that increased from 2.5 weeks to 2 months, and declined at 7 months, whereas, immunization with HKCC adjuvant induced M-specific IgA titers that declined at 7 months (**Figures 6C**, II).

### LP_Mix_ immunizations elicited a robust proliferation response in splenocytes and bone marrow cells upon *ex vivo* stimulation with recall antigens (P_1-7_)

Clonal expansion of antigen-specific lymphocytes, both primary and memory cells, is essential for generating an effective adaptive immune response (48). Here, we assessed the recall responses of splenocytes and bone marrow cells, derived from LP_Mix_ immunizations, against individual peptides (P_1-7_), at 2.5 weeks, 2 months, and 7 months.

**Figure 7:**
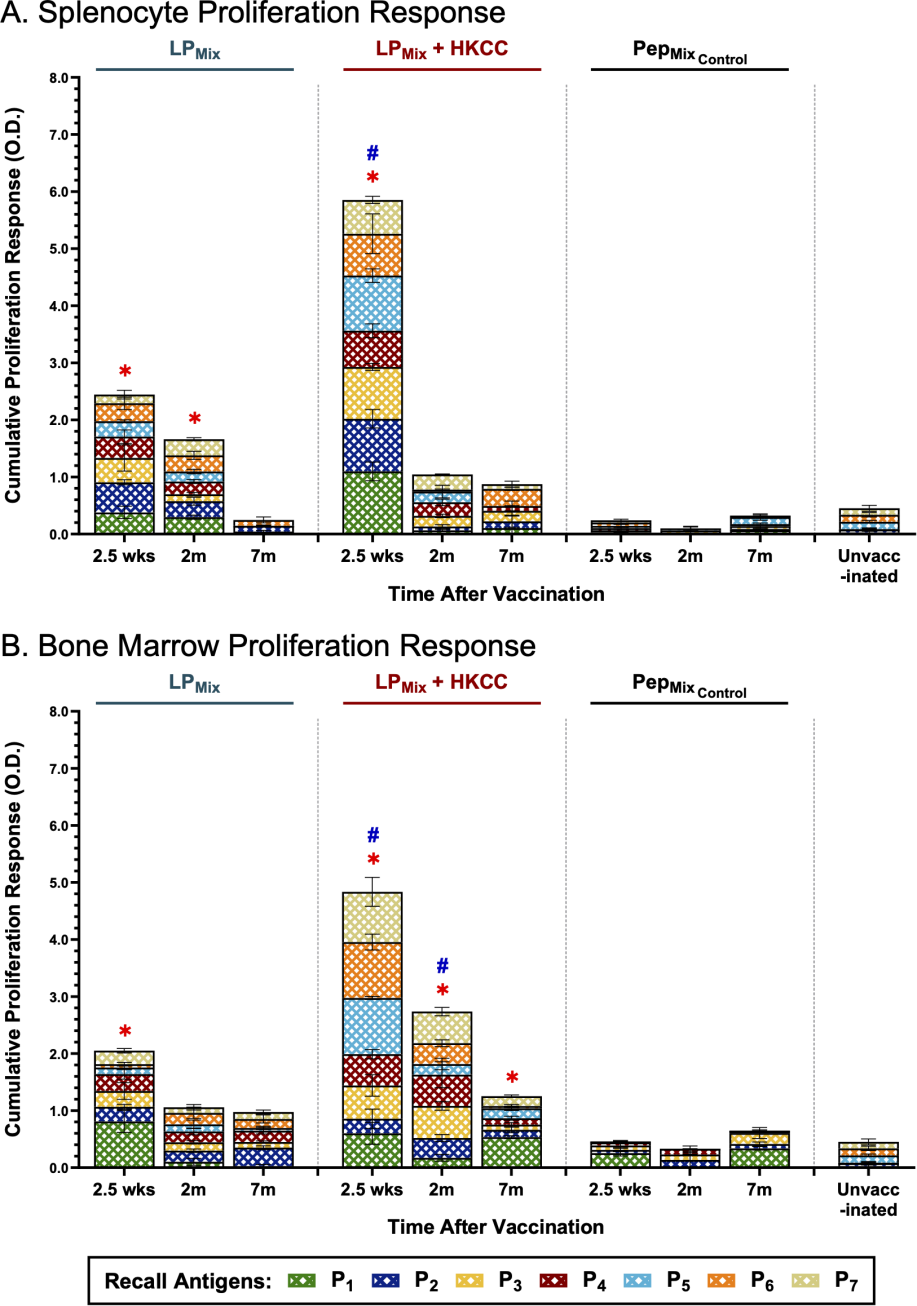
Intranasal immunizations with LP_Mix_ groups induce long-term antigen-specific proliferation responses in the spleen and bone marrow. Male C57BL/6 mice (n=5 per immunization group) were immunized intranasally (D-14, D0) with LP_Mix_ groups. At the 2.5 week, 2 month, and 7 month timepoints, **(A)** splenocytes and **(B)** bone marrow cells derived from LP_Mix_-immunized mice, were cultured with irradiated APCs from unvaccinated syngeneic mice, and re-stimulated *ex vivo* with individual peptides (P_1-7_; crosshatched bars), at 0.1 µg/mL for 4 days. Cell proliferation was determined by BrdU incorporation in proliferating cells. Mice immunized with the corresponding peptide mix vaccine formulation, without the palmitoyl moiety, (Pep_MixControl)_, and unvaccinated mice, were used as controls. Data are represented as mean ± SEM of triplicate cultures, from two independent repeat experiments. Two-way ANOVA, followed by Tukey’s *post-hoc* test was used to determine significance. * and # indicated significant differences (P ≤ 0.05) between the LP_Mix_-immunized and unvaccinated groups, and the respective adjuvant and non-adjuvant groups, respectively.

When assessing all components of the lipopeptide-mix vaccine, LP_Mix_ immunizations induced the highest splenocyte proliferation responses at the 2.5 week timepoint (**Figure 7A**). Moreover, the addition of the mucosal adjuvant, HKCC, significantly increased splenocyte proliferation responses, compared to no adjuvant, and unvaccinated groups (**Figure 7A**). Following the 2.5 week timepoint, splenocyte proliferation declined over time for both LP_Mix_ immunization groups (**Figure 7A**). Despite the decline, the LP_Mix_ immunization induced a higher proliferation response at 2 months, compared to the unvaccinated group (**Figure 7A**). At the 7 month timepoint, splenocyte proliferation responses were similar to the unvaccinated group (**Figure 7A**). Taken together, LP_Mix_ immunizations induced splenocyte proliferation responses that diminished after 2 months.

Next, LP_Mix_-induced bone marrow proliferation responses were characterized by a declining trend over time (**Figure 7B**). At 2.5 weeks and 2 months, LP_Mix_ + HKCC immunizations induced significantly higher bone marrow proliferation responses, against recall antigens, P_1-7_, compared to LP_Mix_ and unvaccinated groups (**Figure 7B**). Interestingly, at 7 months, only LP_Mix_ + HKCC immunizations demonstrated a bone marrow proliferation response that was significantly higher than the unvaccinated groups (**Figure 7B**). All in all, despite the declining trend, the addition of the HKCC mucosal adjuvant significantly increased the proliferation responses against recall antigens, P_1-7_, and the longevity of the response up to 7 months (**Figure 7B**).

Notably, the corresponding peptide mix immunization group, Pep_MixControl_, induced a splenocyte and bone marrow proliferation response that was comparable to the unvaccinated group (**Figures 7A, B**). Evidently, a comparison between the LP_Mix_ and Pep_MixControl_ immunization groups indicates that the addition of the palmitoyl moiety enhances antigen-specific proliferation responses in the spleen and bone marrow.

### LP_Mix_ immunizations induced long-term T cell memory responses in the lungs, spleen, and bone marrow.

Investigating the cellular arm of the immune system, we assessed the frequency and expression profiles of LP_Mix_-induced CD4^+^/CD8^+^ memory T cells, in the lungs, spleen, and bone marrow, for 7 months after the 2^nd^ immunization. Cells were stimulated *ex vivo* with a pool of peptides (1ug/ml of each peptide, *in vitro*) to determine overall antigen-specific stimulation of those cell populations.

**Figure 8:**
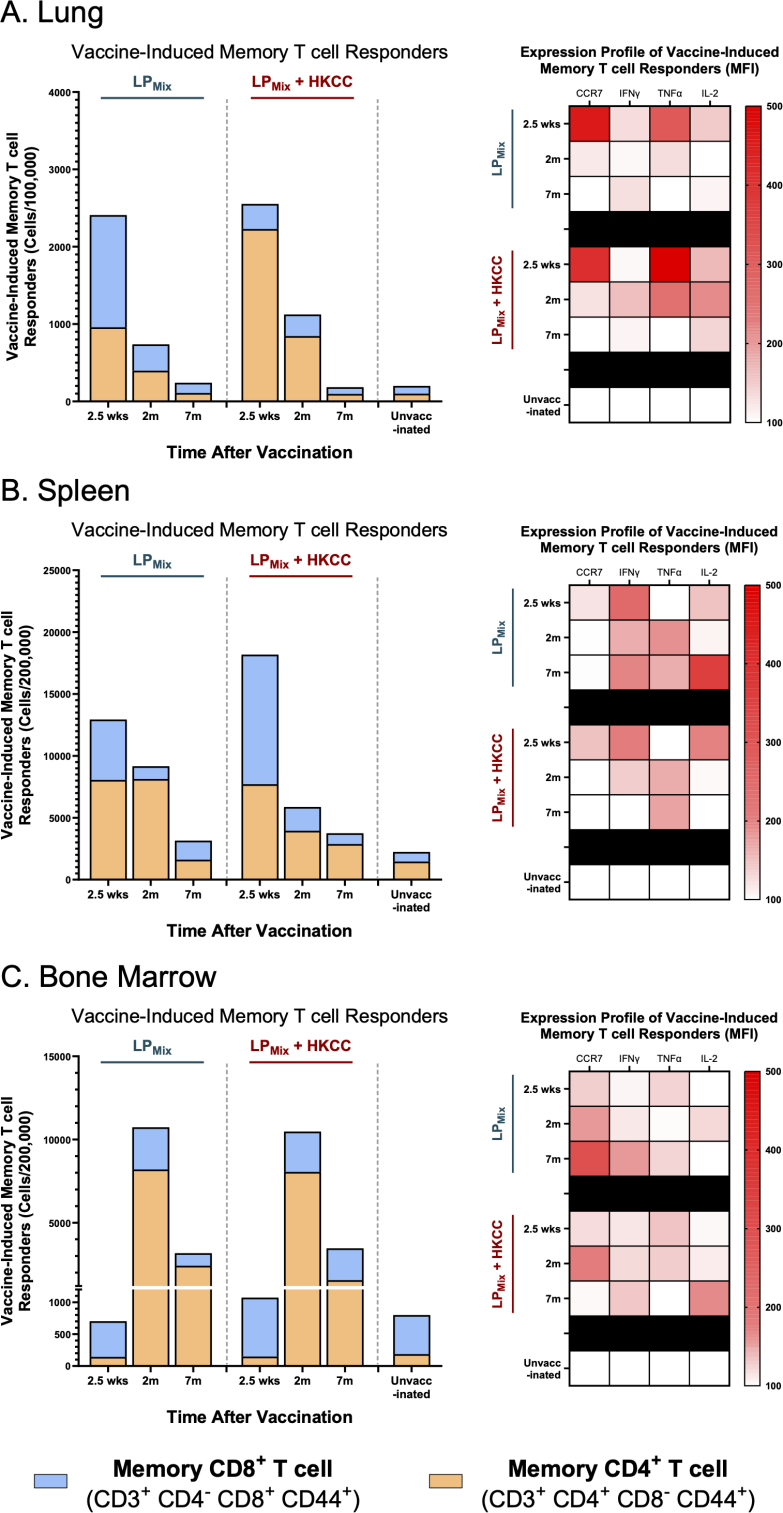
LP_Mix_ immunizations induce long-term T cell memory responses in the lungs, spleen, and bone marrow. Male C57BL/6 mice (n=5 per immunization group) were immunized intranasally (D-14, D0) with LP_Mix_ groups. At the 2.5 week, 2 month, and 7 month timepoints, and cell counts of memory CD4^+^ T cells (CD3^+^ CD4^+^ CD8^-^ CD44^+^; Orange), and CD8^+^ T cells (CD3^+^ CD4^-^ CD8^+^ CD44^+^; Blue) were determined in the **(A)** lungs, **(B)** spleen, and **(C)** bone marrow. Bar graphs summarized the mean number of memory CD4^+^/CD8^+^ T cells, among LP_Mix_ immunization groups. Heatmaps showed the expression of CCR7, IFNγ, TNFα, and IL-2 of the memory T cell population (CD3^+^ CD44^+^), for each LP_Mix_ immunization groups, at the 2.5 week, 2 month, and 7 month timepoints. Data represent two independent, repeat flow cytometry experiments.

In the lungs and spleen, vaccine-induced memory T cells declined over time, for the LP_Mix_ and LP_Mix_ + HKCC immunization groups (**Figures 8A, B**). Whereas, in the bone marrow, the memory T cell populations expanded from 2.5 weeks to 2 months, followed by a decline at 7 months (**Figure 8C**). Notably, despite this decline, LP_Mix_-induced bone marrow memory T cell counts were higher compared to the unvaccinated group, and their respective LP_Mix_ immunization group at 2.5 weeks (**Figure 8C**). Furthermore, the bone marrow memory T cells consisted of predominantly CD4^+^ memory T cells, at 2 months and 7 months (**Figure 8C**). Taken together, as memory T cells declined in lungs and spleen over time, the bone marrow memory T cell counts demonstrate the persistence and maintenance of memory T cell responses induced by LP_Mix_ immunizations.

Next, looking at the expression profiles of the memory T cell populations in the lungs, LP_Mix_ immunizations demonstrated maximal effector functionality at the 2.5 week timepoint, characterized by an increase in CCR7, IFNγ, TNFα, and IL-2 (**Figure 8A**). Following the 2.5 week timepoint, the expression of these effector molecules in T cells were substantially lowered in the LP_Mix_ immunization group, whereas the LP_Mix_ + HKCC immunization group maintained expression of IFNγ, TNFα, and IL-2, up to 2 months (**Figure 8A**). Overall, memory T cells induced by LP_Mix_ + HKCC immunizations were able to maintain effector functionality for a longer time in the lungs, compared to the LP_Mix_ immunizations.

In the spleen, LP_Mix_ and LP_Mix_ + HKCC immunizations induced memory T cells that showed similar expression of effector molecules at 2.5 weeks and 2 months, characterized by high expression of CCR7, IFNγ, IL-2, and TNFα, respectively (**Figure 8B**). At 7 months, LP_Mix_-induced splenic memory T cells showed higher expression of IFNγ and IL-2, compared to LP_Mix_ + HKCC (**Figure 8B**). As high levels of IL-2 have been shown to drive T cell differentiation into short-lived effector T cells and the induction of effector molecules (IFNγ and TNFα), LP_Mix_ immunizations shows an effector phenotype (**Figure 8B**) (49).

Furthermore, in the bone marrow, LP_Mix_ immunizations induced memory T cells that increased in CCR7 expression from 2.5 weeks to 2 months (**Figure 8C**). Thereafter, the expression of CCR7 continued to increase for the LP_Mix_ group, whereas memory T cells induced by the LP_Mix_ + HKCC immunizations showed decreased expression of CCR7, while increasing the expression of IL-2 and IFNγ (**Figure 8C**). CCR7-deficient memory T cells have shown to support CD4^+^/CD8^+^ memory T cell development, promote homeostatic proliferation, displaying a memory phenotype, thus LP_Mix_ + HKCC immunizations induced a better memory phenotype (50).

## Discussion

The COVID-19 pandemic was downgraded from a public health emergency in May of 2023, however, due to continuing infections with novel SARS-CoV-2 variants, COVID-19 remains an ongoing concern. Current vaccines, though initially effective, induce short-lived immunity and show compromised efficacies against emerging VOCs and partly due to immune imprinting from frequent booster doses (4, 51). Therefore, there is a pressing need for a next-generation vaccine candidate that provides durable and long-lasting protection against a broad-spectrum of CoVs, and protective mucosal immunity. Previously, we reported on a LP_Mix_ vaccine construct, which consists of seven highly conserved lipopeptides derived from the SARS-CoV-2 S-, N-, and M-proteins (43). The LP_Mix_ vaccine induced early and robust CD4^+^/CD8^+^ T cells, antigen-specific B cells, and cross-reactive IgM/IgA antibody responses against multiple SARS-CoV-2 VOCs (44). Moreover, the LP_Mix_ vaccine showed efficacy against the Omicron (BA.5) variant in a Golden Syrian hamster infection model (44). In the current study we demonstrate that LP_Mix_ immunizations elicited durable, long-lasting systemic IgM/IgG, and mucosal IgA antibodies against multiple SARS-CoV-2 VOCs, SARS-CoV-1 (Tor2), MERS-CoV, and HCoVs, and also showed neutralizing capabilities (**Figures 2**, **4**, **5**). Also, N- and M-specific antibody titers were generated in the lung mucosa and systemic blood (**Figure 6**). The LP_Mix_ vaccine induced not only a robust splenocyte response but also an antigen-dependent bone marrow proliferation response leading to the generation of long-lived antigen-specific B cells, and memory CD4^+^/CD8^+^ T cells in the lungs, spleen, and bone marrow (**Figures 3**, **7**, **8**). Furthermore, these responses were maintained for up to 7 months after the 2^nd^ immunization in mice, which is estimated to be equivalent to 20–30 years in humans (52). The obtained results are summarized in **Tables 1** and **2**.

**Table 1:** Summary of vaccine-induced antibody responses.

Immunization Groups		LP_Mix_			LP_Mix_ + HKCC		
Timepoints		2.5 weeks	2 month	7 month	2.5 weeks	2 month	7 month
Serum Antibody Responses							
SARS-CoV-2 Cross-Variant Responses	IgM	+	+++	++	+	++	++++
	IgG	++	++	++	++	++	+
Heterologous CoV Responses	IgM	+	++	++	++	++	++++
	IgG	++	++	+++	++	+	++
Neutralization Antibody Responses		++	++	++	+++	+++	+
Nucleocapsid (N)-Specific Responses	IgM	+	++	++	+	++	++++
	IgG	-	-	-	-	-	-
Membrane (M)-Specific Responses	IgM	+	++	+++	+	++	++++
	IgG	+	++	++	+	++	++
BAL Antibody Responses							
SARS-CoV-2 Cross-Variant Responses	IgA	+++	++	+	+++	++++	+
Heterologous CoV Responses	IgA	++	+	-	+++	+++	-
Neutralization Antibody Responses		++	++	+	+++	++	+
Nucleocapsid (N)-Specific Responses	IgA	+	+	+	+++	+	+
Membrane (M)-Specific Responses	IgA	++	+++	++	++	++	+

For each immune parameter, mean values were considered from each immunization group for each timepoints shown in the table, and rated using the following notation: Symbol “ + ” denoted; Strongest (++++), Strong (+++), Moderate (+++), Weak (++), and weakest (+) magnitude of the response; Symbol “ - ” denoted responses similar to the unvaccinated group.

**Table 2:** Summary of vaccine-induced responses in the lungs, spleen, and bone marrow.

Immunization Groups	LP_Mix_			LP_Mix_ + HKCC		
Timepoints	2.5 weeks	2 month	7 month	2.5 weeks	2 month	7 month
Lung Immune Responses						
Omicron(BA.1)-Specific IgM^+^ B cells Responses	**++++**	**+++**	**+**	**++++**	**++++**	**++**
Memory CD4^+^ T cells Responses	**+++**	**+**	**-**	**++++**	**+++**	**-**
Memory CD8^+^ T cells Responses	**+++**	**+**	**-**	**+**	**+**	**-**
Spleen Immune Responses						
Cumulative Antigen-Specific Proliferation Responses	**+++**	**++**	**-**	**++++**	**+**	**+**
Omicron(BA.1)-Specific IgM^+^ B cells Responses	**-**	**++++**	**+**	**-**	**++**	**+++**
Memory CD4^+^ T cells Responses	**+++**	**+++**	**-**	**+++**	**+**	**+**
Memory CD8^+^ T cells Responses	**++**	**-**	**-**	**++++**	**-**	**-**
Bone Marrow Immune Responses						
Cumulative Antigen-Specific Proliferation Responses	**++**	**+**	**+**	**++++**	**+++**	**+**
Omicron(BA.1)-Specific IgM^+^ B cells Responses	**-**	**+**	**+++**	**-**	**++**	**++++**
Memory CD4^+^ T cells Responses	**-**	**++++**	**++**	**-**	**++++**	**++**
Memory CD8^+^ T cells Responses	**-**	**-**	**-**	**+**	**-**	**-**

For each immune parameter, mean values were considered from each immunization group for each timepoints shown in the table, and rated using the following notation: Symbol “ + ” denoted; Strongest (++++), Strong (+++), Moderate (+++), Weak (++), and weakest (+) magnitude of the response; Symbol “ - ” denoted responses similar to the unvaccinated group.

Vaccine-induced adaptive responses in the lung mucosa can serve to prevent SARS-CoV-2 infections at the primary site and protect against severe lung pathologies. Assessment of mucosal immunity demonstrated that the LP_Mix_ immunizations led to a long-lasting humoral and cellular response in the lungs. Specifically, intranasal immunizations with LP_Mix_ induced cross-reactive IgA titers, demonstrated increased cross-reactive Omicron (BA.1)-specific IgM^+^ B cells, and induced higher frequencies of memory CD4^+^/CD8^+^ T cells (**Figures 2C**, **3A**, **4C**, **8A**). The mucosal lining is the first line of defense against respiratory viruses, which includes antibodies able to opsonize, neutralize, and inactivate incoming pathogens. We showed that LP_Mix_ immunizations induced cross-reactive IgA antibodies against a broad-spectrum of CoVs, including nine SARS-CoV-2 VOCs, SARS-CoV-1, MERS-CoV, and two HCoVs (**Figures 2C**, **4C**). Also, BAL samples showed clinically significant nAb titers, indicating the LP_Mix_ vaccine’s ability to prevent SARS-CoV-2 infections at the site of infection (**Figures 5B**). Many studies have shown that nAb titers protect against SARS-CoV-2 infections and accurately predict vaccine efficacies, however, these titers are short-lived and diminish within 2 months after vaccination and/or natural infection (46, 53, 54). Similarly, LP_Mix_ immunizations induced serum and BAL nAb titers that declined after 2 months, however, despite the decline, these responses were maintained at clinically significant levels, up to 7 months (**Figure 5B**). Concurrent with the long-lasting mucosal IgA and nAb titers, LP_Mix_ immunizations induced Omicron (BA.1)-specific IgM^+^ B cells in the lungs that persisted up to 2 months, followed by decline at 7 months (**Figure 3A**). Ellebedy et al. have shown long-lived, antigen-specific B cells play an essential role in secreting antibodies and maintaining Ig levels in the mucosal lining over time (55). Also, patients who have undergone splenectomy and show depleted levels of IgM^+^ memory B cells demonstrated a reduction in mucosal IgA^+^ plasma cells and a deficiency in secretory IgA in the mucosal lining (56). This highlights the importance of lung and splenic Omicron (BA.1)-specific IgM^+^ B cells induced upon LP_Mix_ immunizations, and their potential role in promoting long-lasting mucosal IgA titers.

In addition to S-based humoral responses, N-/M-specific antibody titers have shown to strongly correlate with protection against consequences of disease, and milder lung pathologies (24, 34, 41). A recent study demonstrated that SARS-CoV-2 infected cells secrete N-protein, obstructing the functionality of 11 human chemokines (57). Therefore, N-specific antibodies can help neutralize viral shedding of the N-protein, while restoring the functionality of chemokines and effective communication between immune cells during SARS-CoV-2 infections. Our study demonstrated that the LP_Mix_ immunizations induced long-lasting IgM and IgA titers against the N-protein that were maintained up to 7 months (**Figure 6**). This suggests that the LP_Mix_ immunizations have the potential to produce long-term protection against disease, lung pathologies, and viral shedding of the N-protein. Next, the SARS-CoV-2 M-protein is present on the surface of virally-infected cells, but antibody responses are not neutralizing. Instead, M-specific antibodies function as viral tags that activate antibody-dependent cell-mediated cytotoxicity (ADCC) and cytokine secretion in NK cells, facilitating viral clearance via antibody/Fc-receptor interactions (58). Here, we show that LP_Mix_ immunizations induced M-specific antibody in the lung mucosa (IgA) and systemic blood (IgM/IgG), that persisted up to 7 months (**Figure 6**). Overall, N-/M-specific antibody titers along with Spike-specific antibodies, induced upon LP_Mix_ immunization, can contribute to effective viral clearance.

Along with the humoral responses, the mucosal barrier relies on the cellular arms of the immune system to promote viral clearance and dictates protection from disease during SARS-CoV-2 infections (59). Upon antigen exposure and/or viral challenge, memory CD4^+^/CD8^+^ T cells are characterized by their rapid homing capacities and rapid effector function (60). Notably, lung CD4^+^/CD8^+^ T cell responses have been correlated with reducing viral loads, resulting in milder disease and promoting vaccine efficacy (59, 61). Moreover, LP_Mix_ immunizations induced memory CD4^+^/CD8^+^ T cells that maintained high cell numbers and effector functionality up to 2 months, in the lungs (**Figure 8A**). Consistent with the developmental phases of T cell memory responses, the LP_Mix_ immunizations modulated the expansion phase around 2.5 weeks, the contraction phase at 2 months, and by 7 months, the memory phase was well-established (**Figure 8A**) (62). The ability of the LP_Mix_ vaccine to establish and maintain mucosal memory CD4^+^/CD8^+^ T cell populations highlights the successful priming of the mucosal immune system and development of a sustainable T cell memory response. Notably, the development of memory is a process that occurs within secondary lymphoid tissues, therefore, both humoral and cellular memory responses observed at the mucosal barrier are the result of the underlying systemic processes occurring in spleen, induced by LP_Mix_ immunizations (63).

The ability of lymphocytes to recall antigens and undergo clonal expansion is the hallmark of a successful adaptive response. Furthermore, the expansion of antigen-specific T and B cells, upon antigen exposure, is a prerequisite to establishing memory responses (62, 63). Our work demonstrated that the LP_Mix_ immunizations led to the expansion of splenocytes, including T and B cells, in response to recall antigens from the SARS-CoV-2 S- (P_1_/P_2_), N- (P_3_/P_4_/P_5_), and M- (P_6_/P_7_) proteins (**Figure 7A**). Interestingly, in the spleen, cell counts of memory T cells were consistent with the splenocyte proliferation responses for the LP_Mix_ immunizations (**Figures 7A**, **8B**). Furthermore, the splenic memory T cell population predominantly consisted of the CD4^+^ T cell subset (**Figure 8B**). The role of CD4^+^ helper T cells is to orchestrate the immune response by promoting effector functions of CD8^+^ T cells and stimulating B cells to undergo affinity maturation, isotype switching, and differentiation into long-live plasma/memory B cells (62, 63). LP_Mix_ immunizations induced splenic Omicron (BA.1)-specific IgM^+^ B cells, which were maintained up to 7 months after the 2^nd^ immunizations (**Figure 3B**). In addition, long-lasting serum IgG and mucosal IgA antibody titers were indicative of the presence of long-lived, isotype-switched B cells (**Figures 2**, **4**). Over the 7 months, LP_Mix_ immunizations induced splenic memory T cells, expressing elevated levels of IFN-γ, TNF-α, and IL-2, indicative an effector phenotype (**Figure 8B**). In contrast, LP_Mix_ + HKCC immunizations induced splenic memory T cells that decreased CCR7 and IL-2 expression over time—a phenotype supportive of memory T cell sustainability (**Figure 8B**). Mechanistically, CCR7-deficient memory T cells exhibit enhanced rates of homeostatic turnover, driven by increased exposure to IL-15 compared to IL-7, resulting in better maintenance of the memory populations (50). Notably, the addition of the HKCC mucosal adjuvant enhanced the magnitude of the splenocyte proliferation response and the longevity of memory CD4^+^/CD8^+^ T cells in the spleen (**Figures 7A**, **8B**). The ability of the HKCC mucosal adjuvant to promote dendritic cell (DC) and natural-killer cell (NK) interactions with T cells may be contributing to the longevity of the cellular responses in the spleen, and induction of protective mucosal responses in the lungs (64, 65).

During the 7 months, LP_Mix_ immunizations induced serum IgM and IgG antibody titers that fluctuated in a wave-like pattern—producing repetitive cycles of antibody peaks, followed by troughs, against multiple SAR-CoV-2 VOCs, SARS-CoV-1 (Tor2), MERS-CoV, and HCoVs (**Figures 2**, **4**). Swarthout et al. have previously reported waning of IgG antibody levels, characterized by antibody peaks and valleys, in children under the age of 5 years vaccinated with a 13-valent pneumococcal conjugate vaccine (66). In contrast, several human studies have demonstrated that antibody titers against SARS-CoV-2 VOCs are short-lived and diminish within 6 months after vaccination and/or natural infection (46, 67–69). Interestingly, in examining cross-reactive Ig responses against VOCs over time, it was observed that addition of HKCC with LP_Mix_ induced serum IgM/IgG titers with peaks that successively increased in magnitude over time, without exposure to SARS-CoV-2, possibly due to efficient upstream innate immune stimulation by HKCC and subsequent bolstering of adaptive immunity (**Figure 2**). Notably, IgG responses elicited by LP_Mix_ + HKCC immunizations exhibit a distinct pattern, showing stronger responses against the Delta and Omicron (BA.5) variants compared to the Alpha and Beta variants. It is possible that the epitopes targeted by LP_Mix_ are somehow hidden in the in the recombinant Alpha and Beta variant proteins. However, more broadly, these results suggest that LP_Mix_ + HKCC immunizations, targeting conserved S-epitopes P_1_ and P_2_, may limit immune imprinting and the original antigenic sin phenomenon. With the current mRNA vaccines, targeting the whole-spike protein, and implementing repeated booster shots, IgG responses are skewed—generating higher responses against the original Wuhan strain compared to the novel Omicron variants (70, 71). Comparatively, the patterns observed in the variant-specific IgG responses upon LP_Mix_ immunization highlight that targeting conserved regions of the SARS-CoV-2 structural protein can facilitate the generation of *de novo* humoral responses against novel and emerging SARS-CoV-2 variants. Several studies have shown long-lasting antibody titers are maintained by long-lived plasma cells that reside in specialized bone marrow niche supporting their long persistence (20, 21, 28, 72, 73). Moreover, these long-lived plasma cells constitutively produced antibodies that circulated throughout the body, maintaining long-lasting humoral immunity in the absence of antigen (28, 72, 73).

The goal of vaccination is to induce protective immune responses that can be maintained over the course of a lifetime. For maintaining immunological memory, the bone marrow has been proposed as a reservoir for memory B and T lymphocytes that undergo homeostatic proliferation to maintain cell numbers, promote self-renewal, and upon encountering its cognate antigen, expand into effectors (27, 29, 32, 74). Here, LP_Mix_ immunizations increased bone marrow proliferation responses upon *ex vivo* restimulation with SARS-CoV-2 S- (P_1_/P_2_), N- (P_3_/P_4_/P_5_), and M- (P_6_/P_7_) antigens (**Figure 7B**). Consistent with the splenocyte proliferation results, a declining trend in bone marrow proliferation response was observed. Notably, at 7 months, LP_Mix_ + HKCC immunizations induced antigen-dependent proliferation in bone marrow cells that was significantly higher compared to the unvaccinated group (**Figure 7B**). Several studies have shown long-term memory T cells reside in the bone marrow and undergo homeostatic proliferation to promote self-renewal and the maintenance of the memory T cell population (27, 29, 32, 74). Notably, memory T cells have lower proliferative tendencies and more effector functions. This is seen in the long-term bone marrow response and the memory CD4^+^/CD8^+^ T cell profiles induced by LP_Mix_ + HKCC immunizations as bone marrow memory CD4^+^/CD8^+^ T cells showed a loss of CCR7 expression and an increase in IL-2 and IFNγ expression (**Figures 7B**, **8C**) (62). Next, regarding humoral immunity in the bone marrow, LP_Mix_ immunizations induced long-lasting, antigen-specific B cells, characterized by an increase in B cell surface Ig binding to the Omicron (BA.1) S-protein and surface expression of IgM (**Figure 3C**). Several studies have shown that a specialized eosinophil/stromal cell niche, maintained by survival factors IL-5, IL-6, and APRIL, promote the longevity of antibody-secreting plasma cells in the absence of its relevant antigen (31, 75–78). Considering the bone marrow is highly vascularized, we speculate that these long-lived, antigen-specific B cells may be contributing to the long-lasting systemic IgM/IgG, and possibly mucosal IgA titers observed with LP_Mix_ immunizations. Moreover, the observed wave-like pattern in serum IgM/IgG titers, may be reflective of the turnover rate and varying half-lives of bone marrow plasma cells, within the eosinophil/stromal cell niche (31, 75–78). Similarly, memory T cells continuously circulate throughout the body, including the blood and peripheral tissues, therefore, long-term immunity induced by LP_Mix_ immunizations in the lungs and spleen may stem from the bone marrow’s ability to promote the survival and homeostatic maintenance of memory lymphocytes. The exact mechanism how mucosal immunization with LP_Mix_ with or without HKCC adjuvant allows the development of long-lived memory cells in bone marrow, remains to be deciphered. The bone marrow is a reservoir for long-term immunological memory; understanding the means and mechanisms to optimizing the quality and duration of bone marrow memory responses will help develop future vaccines with long-term protective immunity and reveal the associated immune correlates of protection.

## Materials and methods

### Synthetic lipopeptides, peptides, adjuvants

Synthetic lipopeptides [LP_1_ (Spike S_1_ aa_492-505_): LQSYGFQPTNGVGYK(Palmitoyl)G, LP_2_ (Spike S_2_ aa_814-826_): KRSFIEDLLFNKVK(Palmitoyl)G, LP_3_ (NCAP aa_358-372_): IDAYKTFPPTEPKKDK(Palmitoyl)G, LP_4_ (NCAP aa_317-331_): MSRIGMEVTPSGTWLK (Palmitoyl)G, LP_5_ (NCAP aa_158-172_): VLQLPQGTTLPKGFYK(Palmitoyl)G, LP_6_ (Mem aa_98-112_): ASFRLFARTRSMWSFK(Palmitoyl)G, and LP_7_ (Mem aa_34-48_): LLQFAYANRNRFLYIK(Palmitoyl)G] and their corresponding peptides [P_1_: LQSYGFQPTNGVGY, P_2_: KRSFIEDLLFNKV, P_3_: IDAYKTFPPTEPKKD, P_4_: MSRIGMEVTPSGTWL, P_5_: VLQLPQGTTLPKGFY, P_6_: ASFRLFARTRSMWSF, and P_7_: LLQFAYANRNRFLYI, respectively] were custom synthesized by Genscript Inc. (NJ, USA) with >96% purity (43). Lipopeptides and peptides were stored in DMSO at 10mg/ml, at -20°C, and diluted in PBS or culture medium prior to use. Furthermore, Heat-killed *Caulobacter crescentus* (HKCC), an innate immunity stimulant, was used as an adjuvant (44).

### Heat-killed *Caulobacter crescentus*

*Caulobacter crescentus* (Cc) (ATCC, 19089), was grown as previously described (44). Live Cc was heat-inactivated at 80°C for 60 mins, then centrifuged (6000 rpm for 15 min), and resuspended in PBS. To verify the effectiveness of the heat-inactivated treatment, HKCC was serially diluted and plated on PYE agar plates. For immunizations, only batches that showed no bacterial growth were used.

### Mouse immunizations

All animal studies were conducted at the pathogen-free animal facility (HSLAS) at the University of Alberta in accordance with the guidelines of the Canadian Council on Animal Care (CCAC). All procedures performed on animals in this study were in accordance with regulations and guidelines reviewed and approved in animal use protocol AUP00000212 by the University of Alberta’s Animal Care and Use Committee (ACUC) for Health Sciences.

Four to six-week-old male C57BL/6 mice (purchased from Charles River Laboratory) were immunized twice, 14-days apart, intranasally with a lipopeptide mix vaccine (LP_Mix_: LP_1_, LP_2_, LP_3_, LP_4_, LP_5_, LP_6_, and LP_7_, each LP given at 10 µg/mouse) in the absence or presence of HKCC adjuvant (**Figure 1**). Mice were euthanized 2.5 weeks, 2 months, and 7 months after the 2^nd^ immunization, and bronchoalveolar lavages (BALs), blood, lungs, spleens, and bone marrow were collected (**Figure 1**). Blood was collected on a monthly basis up to 7 months. All animal experiments were repeated twice, each with n=5 mice/immunization group/timepoint. Unimmunized mice were used as controls.

### Detection of IgM, IgG, and IgA antibodies using enzyme-linked immunosorbent assay

NUNC MaxiSorp 96-well flat bottom plates (Thermo ScientificNunc™) were coated with SARS-CoV-2 N-, M-, and S-proteins of the Alpha (B.1.1.7), Beta (B.1.351), Delta (B.1.617), Omicron (BA.1, BA.5, BQ1.1, and EG.5), HCoV-OC48, HCoV-299E, MERS-CoV, and SARS-CoV-1 (Tor2) variants (Genscript Inc., NJ, USA), in individual plates, at 1 µg/mL in PBS. Next, the detection of IgM and IgG in serum, and IgA in BAL was performed according to the antibody ELISA protocol previously described (44). Diluted serum (1:100) and BAL (1:2) samples were performed in triplicates. Plates were read using a DTX 880 Plate Reader (Beckman Coulter, CA, USA) at 405nm, 1.5h after the addition of p-Nitrophenyl Phosphate (PNPP substrate; Southern Biotech., USA). For each antibody isotype, Ig standard curves were performed to interpolate optical density (O.D.) readings to antibody concentrations (pg/mL). Bars are expressed as the mean [Ig] ± standard error mean (SEM) of triplicate wells.

### Neutralizing antibody titers

Using a cPass SARS-CoV-2 Neutralizing Antibody Detection Kit (GenScript Inc., USA), serum and BAL samples from immunized groups were tested for neutralizing antibody titers. The assay was conducted according to the manufacturer’s instructions. Each sample was performed in triplicate, with serum and BAL samples diluted at 1:9 and 1:1, respectively. Plates were read using a DTX 880 Plate Reader (Beckman Coulter, CA, USA) and percent inhibition values were calculated using the formula, below. Bars represented mean percent inhibition ± SEM of triplicate wells.


$$\text{Percent Inhibition }=\left(1-\frac{\text{O}.\text{D}.\text{ value of sample }}{\text{O}.\text{D}.\text{ value of }\left(-\right)\text{ Control}}\right)\;\times \;100\%$$

### Antigen-specific proliferation assay for splenocytes and bone marrow cells

Single-cell suspensions of spleen and bone marrow cells were acquired using previously described Bio-protocols (79, 80). Antigen-specific proliferation assays were performed as described previously (43). Splenocytes and bone marrow cells derived from immunization groups were re-stimulated *ex vivo* with recall antigens, P_1_-P_7_, at 0.1 µg/mL, and incubated for 4 days at 37°C. Next, using a Roche Cell Proliferation ELISA, BrdU colorimetric kit (Sigma-Aldrich, MO, USA), cell proliferation was measured and O.D. readings were acquired using a DTX 880 Plate Reader (Beckman Coulter, CA, USA), set at 450nm. O.D. readings were subtracted by background and represented as mean O.D. ± SEM of triplicate values.

### Flow cytometry analysis of long-term memory B cell and T cells

From each immunization group, single-cell suspensions of the lungs, spleens, and bone marrows were acquired (79, 80). Isolated lung lymphocytes (2x10^6^ cells), splenocytes (2x10^6^ cells), and bone marrow cells (2x10^6^ cells) were plated in a flat bottom 96-well plate. For T cell stimulations, cells were cultured with a peptide pool of P_1-7_ (each peptide at 1 µg/mL) for 4 days at 37°C, followed by the addition of PMA (50 ng/mL) and ionomycin (500 ng/mL) for 20h at 37°C. Next, Brefeldin A (1.5 µg/mL) was added 4h before staining with a T cell panel (Live/Dead NIR, CD3-Alexa Fluor 488, CD4-BV786, CD8b-BUV615, CD69-PE-Cy7, CD62L-BUV805, CD44-eFluor450, CCR7-BV650, IFNγ-PE, TNFα-BV711, IL-2-BUV737, IL-10-PerCP-Cy5.5, and Foxρ3-APC; Thermo Fisher Scientific, UK), using an established flow cytometry staining protocol (81). For B cell stimulation, cells were stimulated with soluble anti-IgM F(Ab’)_2_ (20 µg/mL), CpG-oligodeoxynucleotides (500 ng/mL), IL-4 (10 ng/mL), and CD40 (1 µg/mL) in a 37°C incubator, for 6 days. This was followed by staining with a B cell panel, which included Live/Dead NIR, CD19-BUV615, IgD-PE-Cy7, IgM-SB780, and Omicron (BA.1) Spike protein-APC conjugated (Thermo Fisher Scientific, UK). Samples were run on the Cytek^®^ Aurora Spectral Flow Cytometer (Cytek^®^ Biosciences, USA) and analyzed using the FlowJo v10.10 software.

### Graphs and statistical analysis

Data was analyzed and graphed using GraphPad Prism Software 10.2.3 (CA, USA). Statistical significance was determined using a two-way ANOVA, followed by a multiple comparisons test for the proliferation assays, antibody ELISAs, and neutralizing antibody assays. A P ≤ 0.05 was used to determine significance.

## Data Availability

The original contributions presented in the study are included in the article/supplementary material. Further inquiries can be directed to the corresponding author.
